# COVID-19 Vaccines in the Pediatric Population: A Focus on Cardiac Patients

**DOI:** 10.1155/2024/2667033

**Published:** 2024-05-15

**Authors:** Ghena Lababidi, Hossam Lababidi, Fadi Bitar, Mariam Arabi

**Affiliations:** ^1^Faculty of Medicine, American University of Beirut Medical Center, Beirut, Lebanon; ^2^Children's Heart Center, Division of Pediatric Cardiology, Pediatric Department, American University of Beirut Medical Center, Beirut, Lebanon

## Abstract

Due to the deleterious global impact of the COVID-19 pandemic, tremendous effort has been invested in the development of vaccines against the virus. Vaccine candidates are first tested in adult populations, a number of which have been approved for EUL by the WHO, and are in use across the USA and MENA region. The question remains whether these (or other) vaccines should be recommended to a neonatal, pediatric, and/or adolescent cohort. Incidence and severity of COVID-19 infection are low in pediatric, neonatal, and adolescent patients. Since both overall incidence and severity are lower in children than in adults, safety is an important consideration in vaccine approval for these age groups, in addition to efficacy and a decreased risk of transmission. The following review discusses vaccine immunology in children aged 0–18 years, with emphasis on the negative impact of the COVID-19 pandemic on the lives of children, considerations for pediatric vaccine approval, and available vaccines for pediatric cohorts along with a breakdown of the efficacy, advantages, and disadvantages for each. This review also contains current and future perspectives, as well as a section on the cardiovascular implications and related dynamics of pediatric COVID-19 vaccination.

## 1. Introduction: The COVID-19 Pandemic—An Overview

The COVID-19 pandemic has been unprecedented in terms of its impact on various sectors of society. Not only is the virus deadly in and of itself, but the pandemic has overwhelmed healthcare systems around the world, leading to increased mortality due to other causes. Models suggest that over a 6 month period between 2020 and 2021, the effects of this pandemic on the ability of healthcare systems in developing countries to cover childbirth interventions lead to at least 253,500 extra child deaths and 12,200 extra maternal deaths [1]. In addition, the pandemic's effects on the global economy and personal financial security have been notable, with the unemployment rate in the United States rising to 14.8% in March 2020 compared to 4% in March 2018 [2]. The effects do not stop at the current generation of adults, as school closure due to COVID-19 has been shown to impact the education and performance of students [3]. Minority and low-income children have been shown to be the most disproportionately and adversely affected by the COVID-19 pandemic [4]. One study found that there was a 1.9 standard deviation difference in mathematics test scores among students in the pandemic cohort compared to those in the prepandemic cohort [5]. However, infection of children with the first strain of COVID-19 was reported to be low. One study published in February 2020 reported that out of 72,314 COVID-19 cases recorded by the Chinese Center of Disease Control, only 2%, or 965 cases, fell within the age group of 0–19 years. In that same study, the case fatality rate in children under 9 years was 0%, while it was 8% in those aged 70–79 years [6]. Nevertheless, the indirect negative effects of the pandemic have been substantial.

**Figure 1:**
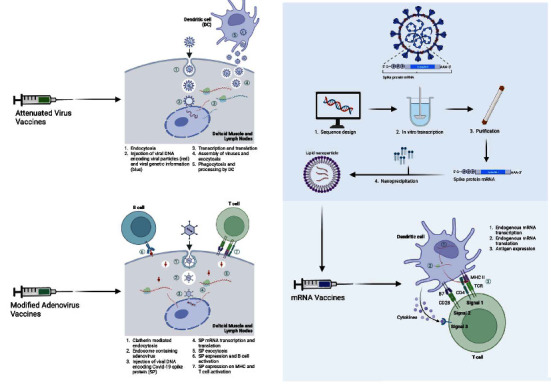
Immunology of various COVID-19 vaccines [8] (this figure was developed using “Biorender” software).

This has, therefore, led to concentrated efforts to halt the progression of the pandemic. Prevention can be split into primary, secondary, and tertiary phases. Research in tertiary prevention has focused on decreasing the morbidity of COVID-19 disease, with various new medications and interventions coming to light. Secondary prevention has focused on early detection, with nasopharyngeal swab and saliva tests with a sensitivity ranging from 83-84.8% coming into play [7]. Still, there is a need for primary prevention. Research focusing on this prevention method has led to the development of several vaccines that have been approved in adult and pediatric cohorts. Vaccine modalities are summarized in Figure 1. The question arises whether these or similar vaccines are efficacious in pediatric patients, and whether the risk outweighs the benefit.

Before considering pediatric and adolescent COVID-19 vaccines, an overview of adult COVID-19 vaccines in use in the United States and the Middle East is included in Table 1.

## 2. Objective

The aim of this literature review is to present all existing vaccines approved for use amongst a pediatric cohort by the WHO and FDA, as well as to summarize data on the safety, efficacy, and immunogenicity of the various vaccine options. This review further summarizes the nature of COVID-19 infection and vaccination in pediatrics and addresses factors promoting vaccine hesitancy. Lastly, this review summarizes the impact of pediatric COVID-19 vaccination on pediatric cardiac patients, as well as discussing the impact of pre-existing cardiovascular diseases on vaccine administration.

## 3. Methods

A literature search was conducted on PubMed under the COVID-19 vaccination MeSH term with results limited to those reported in a pediatric population. The search revealed 3,363 articles between the beginning of 2021 and the end of 2022. A concurrent search was done on clinicaltrials.gov to obtain data on ongoing and future trials relevant to the matter. After title abstract screening, 430 articles were found to be relevant. Papers were only referenced if written in English and published by an official public health authority (CDC, WHO, and so on) or in peer-reviewed journals. A diversity of works was discussed, ranging from observational studies to systematic reviews.

## 4. Results

### 4.1. COVID-19 and Vaccine Immunology in Children

The constitution of the neonatal immune system makes it difficult to assume that vaccines that work in adults would work well in that age group. A neonate derives most of its protection from maternal antibodies, and its adaptive immune system is still rather immature. The main issue to consider is that neonates mount a th2-skewed response compared to other age groups, leading to poor immunogenicity and development of tolerance to foreign antigens at a higher rate than in adults. In addition, B-cells in neonates express decreased levels of coreceptors such as CD80/86 that are required for mounting the T-cell-dependent immune response crucial for lasting immunity. There is also a skew towards B1 cells in this age group, which produce low-affinity IgM antibodies [22]. All in all, neonates produce antibodies that are of lower quality and quantity than those in older infants. Still, the fact that the hepatitis-B vaccine, BCG vaccine, and oral polio vaccine can induce an adequate immune response in this age group is promising [23]. These vaccinations still need to be administered multiple times, as the immature immune system characteristic of this age group is “quick to forget” [23].

The shift from a th2 to a th1-polarized response occurs during the early months of life although some children do not fully undergo this transition until the age of 2 years [24]. By the age of 4, the immune response is viewed to have matured, with a greater ability to mount immune responses that lead to lasting immunity. In fact, the immune response to the Pfizer COVID-19 vaccine has been shown to be similar in children between the ages of 5–11 years and in children 12 years and above [25].

Reviewing how the young immune system responds to active immunization is important as it has many clinical implications. Children and adolescents spend a large portion of their time at school, where they come in close contact with many children and adults. It has been demonstrated in a 2021 study conducted in Ontario, Canada, that the virus easily spread from school children to members of their household [26]. Another study conducted in North Carolina suggests that for every 20 cases of community acquired COVID-19, there is one case of school-related transmission, even with face masks being mandatory [27]. Therefore, preventing the spread of COVID-19 in schools becomes a practical necessity if the pandemic is to be halted and schools are to safely reopen.

The literature reports a decrease in COVID-19 symptom severity in a pediatric cohort compared to an adult cohort across dominant strains of the virus [28–30]. Notably, several countries such as Norway witnessed an increase in GP visits for children infected with the delta variant compared to the omicron variant, with similar figures worldwide, emphasizing the importance of preventive measures [31, 32]. A recent systematic review on COVID-19 symptoms in children revealed an estimate of below 1% for COVID-19-related mortality and a 16% incidence of severe cases. The most commonly reported symptoms were fever and cough, appearing in 57 of the 58 reviewed studies. The definition of severe cases was reported to have varied substantially amongst papers, with some definitions being ICU admission, oxygen therapy, hospitalization, and advanced airway support. Thus, researchers indicated a high risk of bias with a Newcastle–Ottawa score of 4, indicating the importance of guidelines and systematic research on the matter [33]. Seroprevalence of COVID-19 amongst a pediatric cohort has been shown to be comparable to that of unvaccinated adults, with a higher proportion of patients experiencing no symptoms, but less than that of vaccinated adults [34, 35].

Generally, hospital-administered care for pediatric COVID-19 patients includes intravenous fluid and oxygen support, nutritional aid, antipyretics, and electrolyte balance monitoring and maintenance drugs [30]. Use of remdesivir immunomodulators and experimental off-label additional antivirals are reserved for hospitalized patients with severe complications and/or respiratory distress [36, 37]. Patients on immunosuppressants resultant of COVID-19 or other conditions are recommended not to take a COVID-19 vaccine, justifying the careful use of immunomodulators to treat COVID-19 [38].

However, it has been widely reported that infants and neonates show increased vulnerability to severe COVID-19 infection compared to children and adolescents [4, 39]. During the pandemic, a rare pediatric multisystem inflammatory syndrome temporarily associated with COVID-19, defined by the WHO as a multisystem inflammatory syndrome in children (MIS-C), has been reported. Presentation of MIS-C varies, but it generally includes clinical manifestations such as life-threatening shock and Kawasaki-like syndrome [40].

Studies also suggest a long-term antibody response in children compared to adults. The C19.CHILD Hamburg study found that antibody titers amongst the pediatric cohort were 1.75 (*p*  <  0.001) times higher 90 days, 1.38 (*p*=0.01) higher 180 days, and 1.54, (*p*=0.001) times higher 270 days after infection than in the adult cohort. Results may be extrapolatable to postvaccine outcomes, as many vaccines rely on an attenuated virus or sequence from ancestral DNA [41]. Children between the age of 0–9 years are less likely to transmit the infection to a household member compared to adults, while adolescents seemingly exhibit a higher potential [42].

### 4.2. Considerations for Pediatric EUL Vaccine Approval/Recommendation

Existing and/or new vaccines against COVID-19 require testing in a pediatric cohort prior to licensing for emergency use (EUL), partially due to the differences between adult, adolescent, pediatric, and neonatal immunity. Due to the promising results of research into child and adolescent immunology, several pediatric COVID-19 vaccines were considered for EUL. EUL licensing is provided to vaccines that address illnesses posing imminent stress for which existing products have not been useful in eradicating. Vaccines must be manufactured in accordance with good manufacturing practices (GMP) and quality management services (QMS). The FDA follows a similar protocol, although to obtain EUL status, phase III clinical trials with a minimum follow-up time of 2 months including a minimum of 3,000 participants must be evaluated by a team of scientists. Concomitant safety and efficacy data from phase I and II trials are also considered. Approval for vaccine use in the EMR generally follows the WHO recommendations [43].

The COVID-19 vaccine has not yet been deemed a yearly or seasonal vaccine. Yearly studies for vaccine efficacy are currently underway [44–48]. It is anticipated that the WHO, CDC, and FDA may license yearly COVID-19 pediatric vaccines, much like the influenza vaccine, depending on the results of the aforementioned yearly studies and in accordance with the previously summarized guidelines.

### 4.3. Available Pediatric COVID-19 Vaccines

Several vaccines have been the subject of phases 2, 3, 4, and completed clinical trials. A summary of pediatric COVID-19 vaccines can be found in Table 2.

The two most widely used vaccines against COVID-19 in a pediatric cohort are the Comirnaty/BNT162b2 and Spikevax/mRNA-1273 vaccines. mRNA vaccines have the added benefit of providing broader immunity to several tissue tropisms. They are most economically favorable, owing to their low-cost manufacturing although they are expensive to store. mRNA vaccines have only been successfully applied in a pediatric cohort to prevent the COVID-19 pandemic [55]. Studies show that amongst conspiracy theorists, these types of vaccines are found to be the most unacceptable [56].

#### 4.3.1. Comirnaty/BNT162b2

The BNT162b2 vaccine manufactured by Pfizer and BioNTech is authorized for use in individuals aged 6 months and above by the FDA [8] although the American Academy of Pediatrics (AAPs) has not yet revised its recommendations and currently only recommends the administration of the COVID-19 vaccine for individuals aged 5–17 years [57].

In a placebo controlled observer blind study of 2260 adolescents aged 12–15 years recruited from pharmacies across the United States, BNT162b2 had a positive safety and low side-effect profile. Side effects were limited to injection site pain (79%–86%), fatigue (60–66%), and headache (55–65%). No severe adverse reactions were experienced. Compared to a cohort of 16–25 year-old vaccine recipients, recipients between the age of 12–15 years had a 1.76 times (95% confidence interval 1.47–2.10) greater titer of neutralizing antibodies. Vaccine efficacy 7 or more days post double dose vaccination was 100% (95% CI, 78.1–100), with none of the vaccinated participants testing positive for COVID-19 compared to 18 participants from the control group [58]. Amongst adolescents 12–15 years of age, estimated vaccine effectiveness was 59.5% 2–4 weeks after dose 2 and 16.6% during month 2; estimated booster dose effectiveness in adolescents 2–6.5 weeks after the booster was 71.1% [59].

The BNT162b2 vaccine has been approved for use in children between the age of 5–11 years as a two-dose series 3 weeks apart with a booster shot a minimum of 5 months after dose 2 [49]. In a phase 2-3 placebo-controlled trial of 2,268 participants aged 5–11 years across the United States, BNT162b2 had a positive safety and low side-effect profile. No serious vaccine-related adverse events were noted. Compared to a cohort of 16–25 year-old vaccine recipients, recipients between the age of 5–11 years had a 1.04 times (95% confidence interval: 0.93–1.18) greater titer of neutralizing antibodies. 3 vaccinated participants tested positive for COVID-19 compared to 16 participants from the placebo group 7 or more days after the second dose [60]. In a test negative, the case-control study conducted during Omicron variant predominance including more than 120,000 tests, the estimated vaccine effectiveness against symptomatic infection for children 5–11 years of age was 60.1% 2–4 weeks after dose 2 and 28.9% during month 2 after dose 2 [59].

The BNT162b2 vaccine has been approved for use in children between the age of 6 months and 4 years as a primary series of 3 doses (0.2 ml each). The initial two doses are scheduled 3 weeks apart with a booster shot a minimum of 8 weeks after dose 2 [61].

#### 4.3.2. Spikevax/mRNA-1273

In a multinational phase 2-3 placebo-controlled trial of 3,732 adolescents aged 12–17 years, the Spikevax/mRNA-1273 also had a positive safety and low side effect profile. Side effects were limited to injection site pain (93.1 and 92.4%), fatigue (44.6 and 70.2%), and headache (38.5 and 30.2%), which were the same adverse effects reported in the placebo group. No severe adverse reactions were experienced in the experimental and placebo groups. The geometric mean titer ratio of neutralizing antibodies in adolescents compared to young adults was 1.08 (95% confidence interval 0.94–1.24). No positive COVID-19 cases were reported 14 days post double dose vaccination in the experimental group, and 4 were reported amongst the placebo cohort [62].

In a phase 2-3 placebo-controlled trial of 4016 kids aged 6–11 years across the USA and Canada, mRNA-1273 also had a positive safety and low side-effect profile. Side effects were limited to injection site pain, fatigue, and headache with no severe adverse reactions including no incidence of multisystem inflammatory syndrome, myocarditis, or pericarditis. One month after the second dose, children who received a 50 microgram dose of the vaccine had a neutralizing antibody titer of 1610 (95% confidence interval: 1457–1780) compared to young adults who received a 100 microgram dose who had an antibody titer of 1300 (95% confidence interval: 1171–1443). Estimated vaccine efficacy was 88% for infection 14 days or more after the first dose. This study was conducted during the time the B.1.617.2 (delta) strain was the dominant strain [63].

### 4.4. Impact of COVID-19 on Pediatric Cardiac Patients

Though the overall burden of COVID-19 in children is low, certain subsets of this population do have a significantly increased risk of morbidity and mortality, and thus may require special attention in regards to vaccination. Of these groups are children with congenital heart disease (CHD), who have been shown to be more susceptible to life-threatening infections [64]. As detailed in one review by Singampalli et al., pediatric patients affected by congenital heart disease were prone to severe illness after infection with relatively benign viruses. This is considered at least partially due to immune system aberrancies that predispose to more severe inflammatory reactions characterized by greater cytokine release [65]. This is exacerbated by T-cell senescence, which further compromises the patient's ability to fight the infection [66]. Although this may raise concerns about the effectiveness of COVID vaccination in this population, a case report by Wurzel et al. demonstrated a robust immune response in an infant with CHD up to 3 months after initial exposure [66].

Given the increased risk of severe COVID-19 in pediatric CHD patients, some concerns naturally arise regarding the impact of COVID-19 on this population. There have been several studies that highlight the clinical course of such patients. One such study with a good sample size (*n* = 94, 83 pediatric) was conducted in India. Of the 94 patients, 48 (51.1%) required hospital admission, while other multihospital studies have estimated the rate of hospital admission amongst healthy children with COVID-19 at only around 5% [67]. Of those that were admitted, many suffered severe complications such as ARDS (14.5%) and 3 (6.2%) required emergency intervention. In addition, it was found that in-hospital mortality of CHD patients that were COVID-19 positive was much higher than those that were not (27.1% vs 9.1%), highlighting the fact that COVID-19 infection can be quite dangerous in these patients and that the high mortality rate cannot be explained by their pre-existing condition alone. The mortality rate was greater in cyanotic versus acyanotic CHD patients, though the difference was not significant [68]. However, in a study conducted on adults with CHD, it was found that cyanotic heart disease (versus acyanotic) was the most important predicting factor for COVID-19 complications [69]. Another study tracked the course of 7 patients with CHD and COVID-19, all of which suffered acute decompensations, with one mortality in a patient with hypertrophic cardiomyopathy [70]. Furthermore, a retrospective review found that pediatric patients with CHD and COVID-19 had significantly longer hospital stays, complication rate, and mortality rate compared to pediatric COVID-19 patients with no CHD [71]. Several other studies echo these findings [72–74], with one demonstrating that the more severe the heart disease, the worse the outcomes with COVID-19 [75].

## 5. Discussion

### 5.1. Weighing the Evidence: Impact of Vaccination

Characteristics of hospitalized patients who have and have not received the vaccine are well reported [76–79]. St. Joseph's Children's Hospital in Tampa, Florida, reported that over a 6 months postpediatric Pfizer vaccine approval period, most patients aged 12–17 years admitted to the ICU were unvaccinated, and all pediatric patients who developed respiratory distress were unvaccinated [76]. In adolescents aged 12–18 years (445 cases and 777 controls), the BNT162B2 vaccine's effectiveness against hospitalization was 94% (CI 95%: 90–96) and 98% against ICU admission and life support receival. This study occurred during the time when the delta variant was the dominant strain [78]. The restoration of social life and normalcy as well as the deleterious role of the pandemic in propitiating health inequality supports the case in favor of COVID-19 immunization [80–82].

Benefits of immunization for children and adolescents are expected to extend to families and the greater community through herd immunity [77, 79, 83]. A compartmental Susceptible-Exposed-Infectious-Recovered model with an input of data from the UK found that if 60% of the pediatric population aged 5–19 had received at least one dose of a COVID-19 vaccine and adults received a booster shot as per the UK booster program guidelines, there would be a 63% reduction in hospitalizations, a 57% reduction in cases, and a 48% reduction in deaths, compared to adult-only booster vaccination [77]. A cross-sectional study also conducted in the UK suggests that at a low (50/100,000/week) compared to a high (1,000/100,000/week) vaccination rate, there would be an estimated increase of 4,360 hospital admissions and 34 deaths over 16 weeks [79].

Although the incidence and disease severity of COVID-19 in children is low [81, 84, 85], there remains a risk of developing MIS-C of unknown pathogenesis and poorly understood prognosis. The risk of resultant residual cardiac damage is not yet established. Thus, it is reasonable to recommend COVID-19 vaccination to not only interrupt the chain of viral transmission but to also protect against the diagnostic risk of MIS-C.

Some reports have linked the development of MIS-C to COVID-19 vaccination [86, 87], but current evidence indicates that the risk is likely lower than that with organic COVID-19 infection. It is believed that the development of severe MIS-C in pediatric patients is associated with an increase in serum levels of receptor binding protein (RBP), which has been shown to occur after both natural COVID-19 infection and mRNA COVID-19 vaccination. A case report by Jain et al. described two cases of MIS-C in pediatric patients within a week of mRNA COVID-19 vaccination at the Children's Hospital of Richmond at Virginia Commonwealth University. Both cases were mild, with rapid improvement upon initiation inpatient treatment [88]. Zambrano et al. conducted a case-control study which found that a diagnosis of MIS-C following exposure to COVID-19 was inversely associated with vaccination, with an odds ratio of 0.22 (95% CI: 0.10–0.52). In addition, 92% of cases were unvaccinated [78]. Another study by Levy et al. revealed that among 33 vaccine-eligible adolescents with MIS-C, none had been vaccinated and 88% were admitted into a PICU, and vaccinated adolescents were found to have a significantly lower risk of MIS-C [89]. COVID-19 vaccination is also well tolerated by pediatric patients with a history of MIS-C, with a study published in late 2022 showing that no children with prior documented MIS-C required subsequent medical evaluation after COVID-19 vaccination [90]. In all, evidence points to COVID-19 vaccination having a net protective effect from severe MIS-C in pediatric patients. While MIS-C is reported to occur after vaccination, it is more likely to be milder than that following organic COVID-19 infection.

Though children in general have a favorable prognosis with COVID-19, the literature shows that those with congenital heart disease are at a greater risk for complications and mortality. Indeed, the British Congenital Cardiac Association released recommendations in 2020 regarding patients that should be considered more vulnerable to COVID-19, with almost all moderate to severe CHD patients qualifying [91]. The CDC already recommends the vaccination of children 6 months and older, but perhaps an even greater emphasis should be put on those with CHD. There is a need for more multicenter studies to further strengthen the association between CHD and COVID-19, as most studies that are currently available have a very small sample size.

### 5.2. Vaccine Hesitancy: Current and Future Perspectives

As of September 21, 2022, the CDC recorded that 1.4 million (8%) children aged 6 months–4 years, 10.7 million (38%) children aged 5–11 years, and 17.6 million (67%) adolescents aged between 12 and 17 years have received at least one dose of a COVID-19 vaccine. Across the United States, pediatric vaccination rates differ from 3%–32% [92]. Vaccine willingness differs drastically around the world [93–95]. Vaccination rates are the lowest in younger age groups, most probably owing to increasing parental vaccine hesitancy.

Several studies report significant vaccine hesitancy [96–98]. One study reports that in a sample of 903 students between the age of 9 and 20, children under the age of 16 and students studying under a lower educational level expressed more vaccine hesitancy [99]. Vaccine hesitancy was also more likely to be observed in people of lower socioeconomic standing [98, 100]. Goulding et al. reported an increased perception of the severity of and risks posed by COVID-19 in 5–11 year-olds postomicron compared to preomicron in focus groups. Authors also reported that of the 67 parents who took part in the focus group, all parents experienced some vaccine hesitancy [96]. Thus, parental acceptance of pediatric COVID-19 vaccinations is critical to their dissemination and necessitates analysis. A study by Mayo Clinic in Rochester Minnesota found that only 30.7% of the parents were somewhat or very likely to vaccinate their children and 39.7% were highly unlikely to vaccinate their children. Of the aforementioned 39.7%, 57% reported that they were not convinced of the efficacy and safety of the vaccine. 58.7% report that more research needs to be done on the matter, underscoring the importance of further trials and testing [101]. Further studies in Saudi Arabia found that although participants had a high score for collective responsibility with respect to vaccine attitudes, they expressed low confidence and a 54% rate of unwillingness to vaccinate their children [102].

Positive predictors of vaccine willingness include high levels of parental anxiety towards the COVID-19 pandemic [103], vaccination according to local schedules [104], and presence of a pediatrician in the immediate family [105]. With the increasing prevalence of vaccine hesitancy, awareness campaigns and patient education are of utmost importance, particularly because a widely reported reason behind vaccine hesitancy is inadequate safety information and worry about side effects [106]. Motivational interviewing by pediatricians during well-child visits were found to reduce vaccine refusal rates by 6.4% compared to a 9% refusal rate preinterview [107]. Thus, pediatricians must navigate guidelines that change day to day in order to make the best recommendation for their patients. Zaveri et al. found that after attending an online education curriculum for pediatric COVID-19 vaccines, physicians reported a statistically significant increase in confidence, except when discussing vaccine risks [97]. This highlights the importance of further studies looking closely and postvaccine complications and contraindications.

The COVID-19 pandemic had a further negative impact on vaccination schedules [84, 108, 109]. A study conducted in Lebanon found a decrease in the utilization of national vaccination services by a factor of 31% in the private sector and 46.9% between February and April 2020. The greatest drop in vaccines was observed for oral polio, hepatitis A, measles, and pneumococcal conjugate vaccines [108]. Postpandemic maintaining vaccination programs particularly for children below the age of 2 years and adolescents will pose a challenge. Vaccinations delayed due to the pandemic should be rescheduled to avoid future public health outbreaks [110].

Improving COVID-19 vaccine immunogenicity in a pediatric cohort has not been significantly investigated. In adult cohorts, stopping systemic immunosuppressive therapy such as methotrexate has been shown to improve immunogenicity with known parallel studies in pediatric age groups [111]. Efforts to improve COVID-19 vaccine efficacy have focused on improving vaccine uptake rates through decreasing vaccine hesitancy rates.

Future perspectives implicate the development of universal vaccines. Allo-priming of healthy elderly patients is currently being discussed as a backup method for pandemic control. Allo-ptiming relies on administering intradermal injections living Th-1-like cells from healthy donors to establish a dominance of allo-specific Th1/CTL memory cells in place of existing aged memory cells. Postviral exposure, primed cells immediately release IFN-ϒ, therefore, initiating a nonspecific immune cascade that limits early viral titers. Release of lysed material from infected cells establishes in-situ vaccination thus initiating a specific immune response to the virus [112]. A phase I/II trial on the safety and efficacy of this treatment modality is currently underway [113].

## 6. Conclusion

mRNA vaccines are the most widely used pediatric vaccines and have been approved for children as young as 6 months old with well-established safety and efficacy profiles. COVID-19 vaccination in pediatric cohorts conveys several benefits chief amongst them a reduction in hospitalization and mortality, as well as the potential preventive effect of the vaccine against MIS-C. Postvaccination incidence of myocarditis has almost entirely been reported as transient and whether it poses significant postvaccination risk is debatable.

## Figures and Tables

**Table 1:** An overview of adult COVID-19 vaccines.

Vaccine name	Vaccine developer and manufacturer	Active ingredient (classical or generational)	Target age group	Dosing	Booster shot:	Approval for use in USA?	Approval for use in EMR?	Efficacy rate
Pfizer/BioNTech comirnaty vaccine (COMIRNATY) [8]	Pfizer/BioNTech	Generational: nucleoside-modified mRNA	5+	2 doses 21 days apart	Yes, for: (i) 65+ (ii) 18+ with high risk of exposure or Hx of previous severe COVID-19	Yes CDC, FDA and WHO approved	Yes approved by WHO for international travel	(i) 91.9% vaccine efficacy against symptomatic COVID-19 [8] (ii) Efficacy against hospitalization ranges between 85% and 100% depending on the consulted study [9–11]

SII/Covishield vaccine [12]	Developer: AstraZeneca/Oxford Manufacturer: Serum Institute of India	Classical: modified adenovirus	18+	2 doses 8–12 weeks apart	Yes, for: (i) 50+ and their contacts (ex: care homes) (ii) High exposure risk individuals (iii) 16+ with high risk of complication (ex: severe asthmatic) [13]	No	Yes approved by WHO for international travel	(i) 72% effective against symptomatic SARS-Cov-2 infection [12] (ii) Larger intervals between doses are associated with greater vaccine efficacy (iii) Efficacy against hospitalization ranges between 70% and 100% depending on the consulted study [14, 15]

AstraZeneca/AZD1222 vaccine [12]	Developer: AstraZeneca/Oxford Manufacturer: SK Biopharmaceuticals, South Korea	Classical: modified adenovirus	18+	2 doses 8–12 weeks apart	Yes, for: (i) 50+ and their contacts (ex: care homes) (ii) High exposure risk individuals (iii) 16+ with high risk of complication (ex: severe asthmatic) [13]	No	Yes approved by WHO for international travel	(i) 72% effective against symptomatic SARS-Cov-2 infection [12] (ii) Larger intervals between doses are associated with greater vaccine efficacy (iii) Efficacy against hospitalization ranges between 70% and 100% depending on the consulted study [14, 15]

Janssen/Ad26.COV 2.S [16]	Johnson and Johnson	Classical: modified adenovirus	18+	1 dose	Yes, for: (i) 18+ Note: a booster is available even if the first dose was not J&J	Yes CDC, FDA and the WHO approved	No Not authorized for use outside the USA, but the WHO approved for international travel	(i) 66.9% efficacy of a single dose against symptomatic moderate and severe SARS-CoV-2 infection [16] (ii) 85.4% efficacy against severe disease and 93.1% efficacy against hospitalization [17]

Moderna COVID-19 vaccine [18]	Moderna	Generational: nucleoside modified mRNA	12+	2 doses 1 month apart	Yes, for: (i) 65+ (ii) 18+ with high risk of exposure or Hx of previous severe COVID-19	Yes CDC, FDA and WHO approved	Yes approved by WHO for international travel	(i) 94.1% efficacy in protecting against symptomatic and severe COVID-19, starting 14 days after the first dose [19]

Sinopharm BBIP COVID-19 vaccine (Sinovac-CoronaVac) [20]	China national Biotec group (CNBG)	Classical: attenuated COVID-19 virus	18+	2 doses 3-4 weeks apart	No Note: Boosters of another vaccine may be given to individuals who took Sinovac-Corona Vac	No	Yes administered in middle East countries such as the UAE	(i) More than 60% efficacy at preventing symptomatic COVID-19 [20]. (ii) Approximate 78% efficacy at preventing hospitalizations due to COVID-19 [21]

**Table 2:** An overview of available pediatric COVID-19 vaccines.

Vaccine name	Vaccine developer and manufacturer	Immunization attributes	Age group	Phase of approval	Advantages	Disadvantages	Examples of similar vaccines currently available
Generational vaccines:							
Pfizer/BioNTech comirnaty vaccine (COMIRNATY) [8, 49]	Pfizer/BioNTech	(i) Express virus antigens *in situ*, thus prompting both humoral and cytotoxic T-cell responses (ii) This generally facilitates a higher caliber of protective efficacy (iii) And prompts an enhanced innate immune response critical for dendritic cell maturation	12–17:	Approved 30 mg, 2 doses 3 weeks apart with booster	(i) Broad range of tissue tropism (ii) Can be rapidly developed (iii) Low-cost manufacturing	(i) Storage conditions are expensive (ii) Bad reputation hinders fast and efficient dissemination	None
			5–11:	Approved 10 mg, 2 doses 3 weeks apart with booster			
			<5:	Approved 0.2 mg, 2 doses 3 weeks apart booster not recommended			
Moderna COVID-19 vaccine (SPIKEVAX) [50]	Moderna		12–17:	Approved 0.5 mL, 2 doses 4–8 weeks apart with booster			
			5–11:				
			<5:	Approved 0.5 mL, 2 doses 4–8 weeks apart without booster			

Protein subunit vaccine							
Nuvaxovid and Covovax [51]	Novavax	Vaccines contain the spike protein of the coronavirus itself formulated as a nanoparticle so as not to cause disease	12–17:	Approved 2 doses 3–8 weeks apart without booster	(i) Cheap and relatively easy to produce (ii) Risk of side-effects usually minimized	(i) Unknown long-term efficacy (ii) May lack PAMPs necessary for stimulating the immune system	(i) Hepatitis B vaccine (ii) Pertussis vaccine
			5–11:	Phase II/II clinical trials in progress in the USA			
			<5:				

Classical vaccines (adenovirus):							
Sinopharm BBIP COVID-19 vaccine (Sinovac-CoronaVac) [52]	China National Biotec Group (CNBG)	A noninfectious antigenic virus incapable of replicating in the host is delivered through a somewhat genetically modified vector	12–17:	Phase II/II clinical trials in progress in the China	(i) Familiar (ii) Systematic and local stimulation of immune response	(i) Slow to develop new vaccines/adapt to new variants (ii) Limitations for use in immunocompromised patients	(i) IPV vaccine (ii) RV vaccine (iii) MMR vaccine (iv) Zoster vaccine
			5–11:	Not in progress			
			<5:				

Classical vaccines (adenovirus):							
Janssen/Ad26.COV 2.S [53]	Johnson and Johnson	(i) DNA encoding the COVID-19 spike protein gene is transfected into a adenovirus 26 (replication incompetent)	12–17:	Phase II/II clinical trials in progress in the USA	(i) Single dose (ii) Storage at refrigerator temperature (iii) Easy to disseminate in	(i) Less effective than double dose vaccines (above)	(i) New experimental measles vaccine (ii) Ad4 and Ad7 used in USA military recruits
			5–11:	Not in progress			
			<5:				
SII/Covishield vaccine [54]	Developer: AstraZeneca/Oxford Manufacturer: Serum Institute of India	(i) The DNA encoding the COVID-19 spike protein gene is isolated and transfected into a replication incompetent chimpanzee common cold virus	12–17:	Phase II/II clinical trials in progress in the UK	(i) Simple to make (ii) Easy to purify to a high titer (iii) Inexpensive to manufacture (iv) Induce B and T-cell responses	(i) Low seroprevalence of adenoviruses in Europe and North America (ii) No reported success of adenovirus vaccines in reducing respiratory illness in children	
AstraZeneca/AZD1222 vaccine [54]	Developer: AstraZeneca/Oxford Manufacturer: SK Biopharmaceuticals, South Korea		6–11:				
			<5:	Not in progress			
